# Surveillance of norovirus among children with diarrhea in four major hospitals in Bhutan: Replacement of GII.21 by GII.3 as a dominant genotype

**DOI:** 10.1371/journal.pone.0184826

**Published:** 2017-09-14

**Authors:** Sonam Wangchuk, Takashi Matsumoto, Hidekatsu Iha, Kamruddin Ahmed

**Affiliations:** 1 Dept. of Microbiology, Faculty of Medicine, Oita University, Yufu, Japan; 2 Royal Centre for Disease Control, Ministry of Health, Royal Government of Bhutan, Thimphu, Bhutan; 3 Dept. of Pathobiology and Medical Diagnostics, Faculty of Medicine and Health Sciences, Universiti Malaysia Sabah, Kota Kinabalu, Sabah, Malaysia; University of Hong Kong, HONG KONG

## Abstract

**Background:**

Diarrhea is a major cause of morbidity and mortality among Bhutanese children. The etiology of diarrhea is not well known due to the challenges of conducting routine surveillance with Bhutan’s modest research facilities. Establishing an etiology is crucial toward generating evidence that will contribute to policy discussions on a diarrheal disease control program. Our previous study, during 2010–2012, revealed that norovirus (NoV) is an important cause of diarrhea among Bhutanese children, and that GII.21 was the major genotype circulating at that time. In other countries, GII.4 is the major genotype responsible for NoV infections. In this update report, we provide new prevalence data to describe the progression of the transformation and distribution of the NoV genotype among Bhutanese children.

**Methods:**

From June 2013 through May 2014, diarrheal stool samples were collected at one national referral hospital in Thimphu, two regional referral hospitals in the eastern and central regions, and one general hospital in the western region of Bhutan. NoV was detected by reverse transcription–polymerase chain reaction (RT–PCR), by amplifying the capsid gene. The RT–PCR results were confirmed by nucleotide sequencing of the amplicons.

**Results:**

The proportion of NoV-positive stool samples was 23.6% (147/623), of which 76.9% were NoV GII and the remainders were NoV GI. The median age of infected children was 15.5 months, with a fairly balanced female: male ratio. NoV GII was most prevalent in the colder months (late November–mid April) and NoV GI had the highest prevalence in the summer (mid April–late September). Nucleotide sequencing was successful in 99 samples of GII strains. The most common genotypes were GII.3 (42.6%), GII.4 Sydney 2012 (15.8%), and GII.4 unassigned (11.9%). No GII.21 was found in any child in the present study. Phylogenetic analysis showed that GII.3 strains in the present study belonged to an independent cluster in lineage B. These strains shared an ancestor with those from different countries and Bhutanese strains circulating during 2010.

**Conclusion:**

NoV remains an important cause of diarrhea among Bhutanese children. Genotype GII.3 from a single ancestor strain has spread, replacing the previously circulating GII.21. Current NoV genotypes are similar to the strains circulating worldwide but are primarily related to those in neighboring countries. NoV GII is prevalent during the cold season, while GI is prevalent during the summer. To develop a NoV infection control policy, further studies are needed.

## Background

Norovirus (NoV) is a single-stranded positive-sense RNA virus with a genome of 7.7 kb. Based on the genetic differences of the capsid protein, NoV is divided into at least six (and sometimes seven) genogroups: GI–GVI. GI and GII are primarily responsible for human infection, although GIV has also been implicated in human diarrhea [1]. With the introduction of commercial rotavirus vaccine in many countries, NoV became the most important cause of childhood diarrhea [2, 3]. Globally and across age groups, NoV is the most common cause of the 685 million diarrhea episodes and 212,000 deaths [4, 5]. NoV is the major cause of community diarrhea among children in developing countries [6]. About 22.7% of diarrhea cases are caused by this virus, and 89% of children experience at least one NoV infection before 24 months of age [7]. An estimated 200,000 deaths occur among children under 5 years of age in developing countries [8].

Bhutan is a small, developing country landlocked between India and China. About 14% of Bhutan’s population is concentrated in the capital Thimphu with the majority scattered sparsely throughout the country. Diarrhea is a major cause of illness and death among Bhutanese children; the morbidity rate from diarrhea in children under 5 years old is 314.6/1,000, while 13% of deaths are attributable to diarrhea [9]. As part of efforts to develop an informed policy for diarrheal disease prevention and vaccination, we initiated a project to estimate the burden of different diarrheal diseases among children under 5 years old in Bhutan. Since the proportion of stools positive for rotavirus and NoV diarrhea in Bhutanese children were 22.3% and 23.7%, respectively, both NoV and rotavirus were deemed equally implicated in infecting Bhutanese children. Our first surveillance of NoV during 2010–2012 showed that GII.21 was the major genotype causing diarrhea in Bhutanese children [10]. The present study was conducted from June 2013 through May 2014 as an update to determine whether there have been changes in the genotype distribution of NoV among the children of Bhutan.

## Materials and methods

### Sample collection

Diarrheal stool samples were collected from children under 5 years of age attending any of the four major hospitals in Bhutan: Jigme Dorji Wangchuk National Referral Hospital (JDWNRH) in the capital, Thimphu; Gelephu Regional Referral Hospital (GRRH) in Gelephu; Phuntsholing General Hospital (PGH) in Phuntsholing; and Mongar Regional Referral Hospital (MRRH) in Mongar. Verbal consent was obtained from each child’s guardian before collecting stool samples. Ethical approval was obtained from the Research Ethics Board of Health, Bhutan (REBH/2010/009). Diarrhea was defined as passing liquid stools at least three times in 24 hrs. Stool samples were collected and stored in the hospital’s freezer at –40°C until they were transported frozen to the Royal Centre for Disease Control, Thimphu, where they were stored at –80°C. The samples were then transferred by commercial courier to the laboratory at the Faculty of Medicine, Oita University in dry ice, where they were stored at –80°C until use.

### Determination of NoV and typing

Stool samples were pretreated with phosphate-buffered solution to a final concentration of 10% (mass/volume). The samples were briefly vortexed, followed by centrifugation at 12000 rpm for 1 min. Supernatant was used for RNA extraction.

RNA from fecal samples was extracted using the QIAamp Viral RNA Kit (QIAGEN, Hilden, Germany); to avoid RNA degradation at each step, DNAse- and RNAse- free plastic wares were used. During RNA extraction, the surface was cleaned by spraying a RNAse decontamination solution followed by a RNase-free water rinse.

NoV was detected by reverse transcription–polymerase chain reaction (RT–PCR) by amplifying the capsid gene at the C region [11]. The RT–PCR results were confirmed by nucleotide sequencing of the amplicons. The nucleotide sequence of the amplicons was determined by the BigDye Terminator v3.1 Cycle Sequencing Kit (Applied Biosystems, Foster City, CA) according to the manufacturer’s instructions and the product was run into the ABI Prism 3100 Genetic Analyzer (Applied Biosystems).

We determined genogroups and genotypes by submitting nucleotide sequences to the Norovirus Genotyping Tool (http://www.rivm.nl/mpf/norovirus/typingtool).

### Phylogenetic analysis

Sequence identity was searched using the Basic Local Alignment Search Tool (BLAST) (https://blast.ncbi.nlm.nih.gov/Blast.cgi), multiple sequence alignment was carried out using ClustalW, and a phylogenetic tree was constructed using Mega 6 [12], applying the neighbor-joining method based on the Tamura–Nei substitution model. For the construction of phylogenetic trees, nucleotide sequences were used (S1 Table). Bootstrap analysis of 1,000 replicates was conducted to determine the significance of the branching of the constructed tree.

## Results

A total of 623 stool samples were collected during the study period, among which 432 (69.3%) were from JDWNRH, 82 (13.2%) from GRRH, 63 (10.1%) from MRRH, and 46 (7.4%) from PGH. The median age of the children was 15 months (range 3–60 months). The study samples included 283 (45.4%) from females and 341 (54.6%) from males. A total of 147 (23.6%) were NoV-positive, among which 34 (23.1%) were NoV GI and 113 (76.9%) were NoV GII. The median age of the NoV-positive children was 15.5 months (range 3–60 months); 73 (49.7%) female and 74 (50.3%) male.

**Fig 1:**
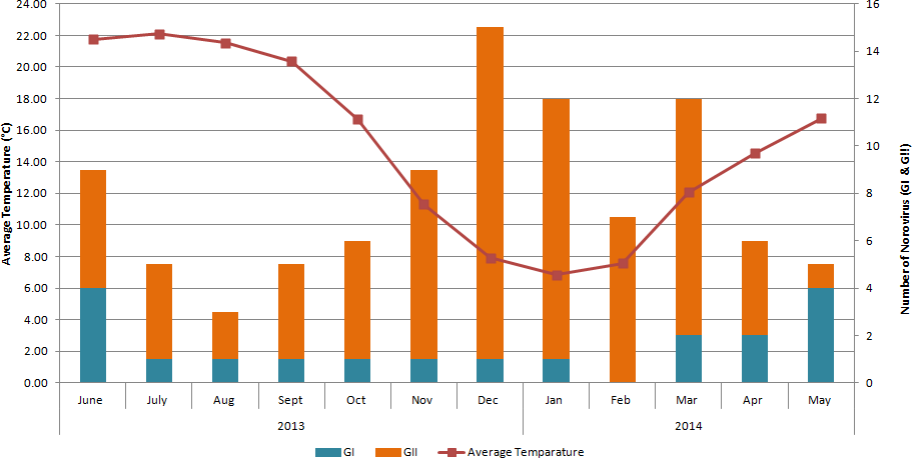
Monthly distribution of norovirus diarrhea cases. Monthly distribution of norovirus diarrhea cases in children in Thimphu. The number of diarrhea cases caused by GII strains was higher during the winter and spring (late November–mid April). The number of diarrhea cases caused by GI strains was higher during the summer (mid April–late September). The left-axis represents the temperature; the right-axis represents the number of norovirus cases.

Whether NoV infection had a seasonal pattern was determined by plotting NoV cases detected during each month in Thimphu. NoV infection was found to be prevalent throughout the year, with a higher prevalence in the winter and spring (late November–mid April), and in the summer (mid April–late September) (Fig 1). NoV GII was the most prevalent strain in the winter and spring (n = 54) compared with the summer (n = 16). However, GI was more prevalent in the summer (n = 11) compared with the winter and spring (n = 5).

Nucleotide sequencing was successful in 99 (87.6%) of 113 GII strains, with genotypes in the following order of prevalence: 43 (43.4%) GII.3; 16 (16.2%) GII.4 Sydney 2012; 10 (10.1%) GII.4 unassigned; 5 (5.1%) GII.15; 4 (4%) GII.13; 3 (3%) GII.6; 2 (2%) GII.17; 2 (2%) GII.22; 2 (2%) GII.2; 1 (1%) GII.14; and 11 (11.1%) undetermined genotypes. GII.3 was dominant in all regions, representing 28/66 (41.2%) in JDWNRH; 3/5 (60%) in PGH; 5/12 (41.7%) in GRRH; and 7/16 (43.7) in MRRH.

**Fig 2:**
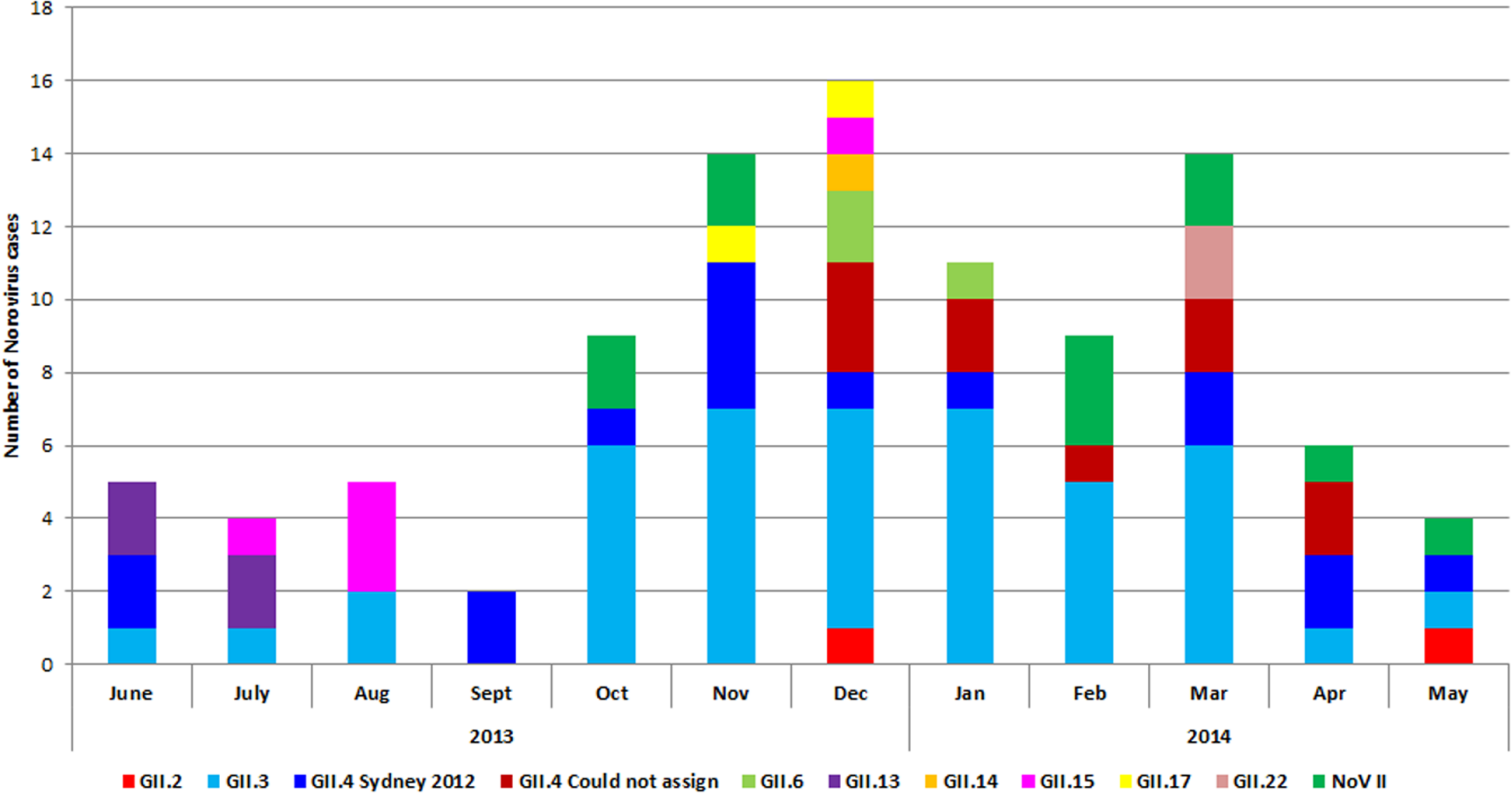
Monthly distribution of norovirus GII genotypes. Graph showing the relationship of different GII genotypes with the months of occurrence. The Y-axis represents the number of norovirus GII genotypes and the X- axis represents the months of occurrence within each year. On the lower part of the graph, each GII genotype is represented by a specific color.

When the genotypes of GII strains were analyzed based on month of detection (Fig 2), no genotype was dominant throughout the year. Rather, genotypes appeared and disappeared in a span of 3–4 months. Different genotypes were prevalent in the same period. Each genotype appeared and then disappeared or maintained a low level (1 case/month). GII.3 was prevalent throughout the study period, maintained a low level (0–2 cases /month) at the beginning then sharply rose in October 2013 (7 cases), maintained a dominance (5–7 cases/month), then sharply decreased from 6 cases/month in March 2014 to a low level (1 case/month). GII.4 Sydney 2012 was prevalent (0–4 cases/month) throughout the study period.

**Fig 3:**
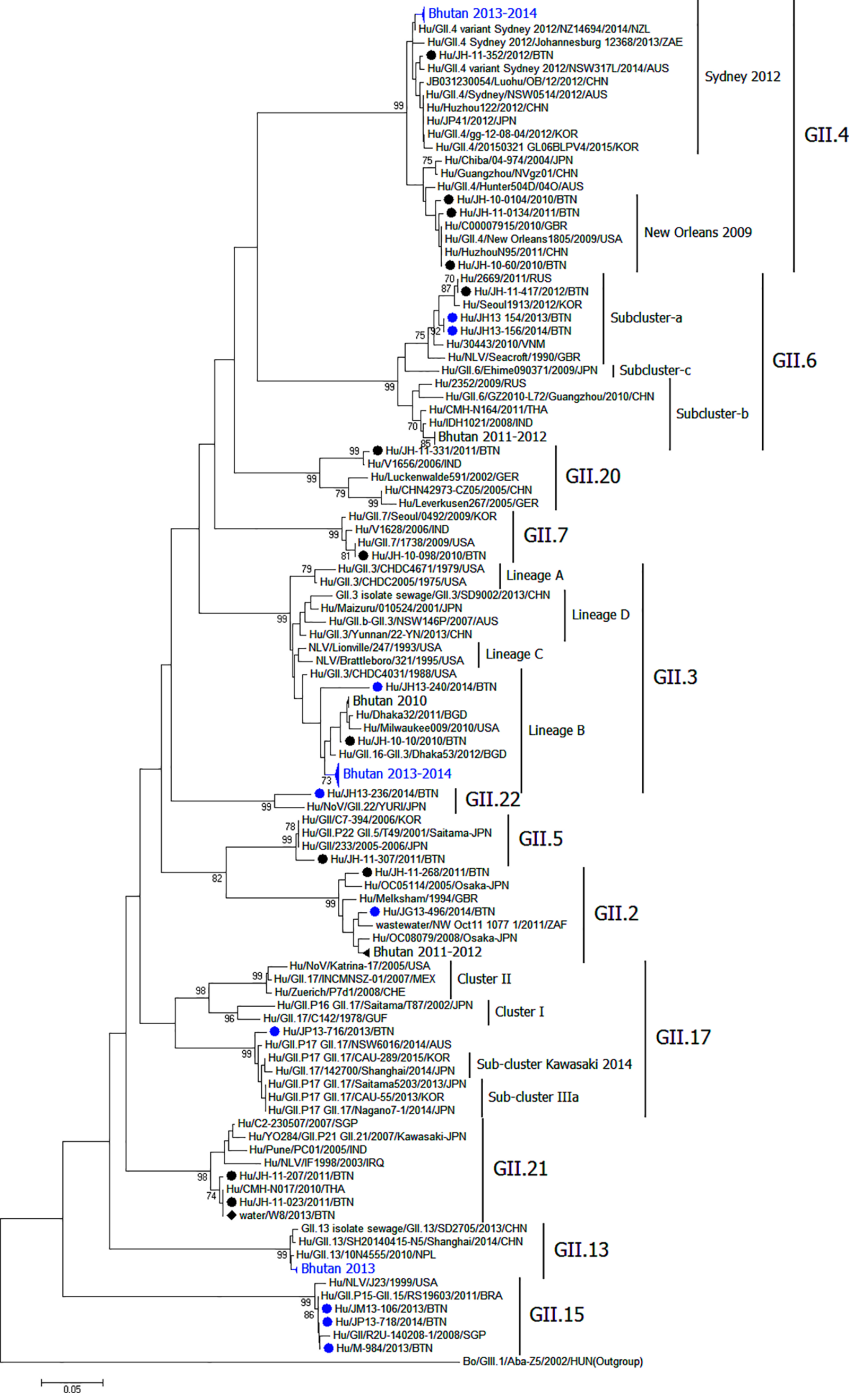
Phylogenetic tree of norovirus GII cases. Phylogenetic tree constructed with the nucleotide sequences of the capsid gene at the C region of norovirus GII strains. Each strain begins with the species name, followed by strain name, year of detection, and country of origin. Bhutanese strains from this and the previous study are preceded by blue- and black-filled circles, respectively. The strain detected in water in the previous study is preceded by a black-filled diamond. Bovine GIII norovirus strain Aba-Z5 was used as an outgroup. The number adjacent to the node represents the bootstrap value. The scale bar shows genetic distance expressed as nucleotide substitutions per site. The DNA Data Bank of Japan/European molecular Biology Laboratory/GenBank accession numbers for the Bhutanese strains of the present study are LC209682–LC209785.

Phylogenetic analysis showed that GII.3 strains in the present study belonged to lineage B (Fig 3). The strains were identical and formed an independent cluster. These strains shared an ancestor with those from Bangladesh, Russia, the USA, and strains circulating in Bhutan during 2010. There were nucleotide substitutions in the VP1 gene of GII.3 strains collected during 2010 and from 2013 to 2014. All 15 strains collected during 2013 to 2014 showed nucleotide substitutions at residues 69 (C to T), 123 (C to T), 162 (C to T) 165 (C to T), and 189 (A to G) compared with the strain collected in 2010.The GII.4 Sydney 2012 in the present study formed a cluster with a strain from New Zealand whereas the strain of 2012 was closer to a strain from Australia. GII.6 strains belonged to subcluster- a, and were closely related to strains from Korea, Russia, and a Bhutanese strain during 2012. However, all Bhutanese GII.6 strains during 2011–2012 belonged to subcluster b. Bhutanese strain GII.22 was closely related to a Japanese strain. Although Bhutanese GII.17 strains were in cluster III, they were outside the Kawasaki subcluster and closely related to strains from Australia and China during 2014 and 2013, respectively. GII.13 strains in the present study were close to strains from Nepal and China. Currently circulating GII.2 strains were close to strains previously circulating during 2011–2012 in Bhutan and strains from Japan. The GII.15 strains from the present study were very close to strains from Singapore and Brazil.

## Discussion

Worldwide, most GII infections are caused by GII.4, followed by GII.3 or GII.6 and then other genotypes, such as GII.2, GII.12, GII.13, GII.17, and GII.7, in varying proportions [12–18]. Contrary to the contemporary concept, NoV GII.21 was the major genotype that caused diarrhea in Bhutanese children during 2010–2012 [10]. The source of infection was suggestive of household tap water, though this could not be identified in streams and water tanks in the Thimphu city water supply sources. The present study revealed that GII.3 replaced GII.21in our study population. However, the prevalence of NoV (23.6%) at this time remains remarkably similar to during the previous study (23.7%), which is similar to other countries [13] and to children hospitalized with diarrhea in other parts of the world [19].

Although the most prevalent NoV genotypes currently responsible for the majority of diarrheal cases are GII.4 and GII.3 [20], there are several differences in their epidemiological pattern. The majority of outbreaks are caused by GII.4, while GII.3 is mostly associated with sporadic NoV infection, particularly in children [21]. In addition GII.3 NoV has been implicated in food-borne outbreaks in both developed and developing countries [21]. Recent studies show that in several countries, GII.4 is the predominant strain causing diarrhea, followed by GII.3 [13–15], while in others, GII.3 is the predominant strain [22, 23]. These results were derived from studies conducted with children.

In 2010, GII.3 was dominant in Bhutan, after which GII.21 continued to dominate for two years [10]. The reason GII.3 is the dominating genotype in Bhutan rather than GII.4 deserves further study. GII.4 and GII.3 genotypes are under differing host-specific selective pressures [21]. GII.4 and GII.3 binding specificity can vary between strains. GII.4 is bound to histo-blood group antigen types A, B, H3 and Le^b^, and Le^y^, while GII.3 is bound to only types A and B strongly and Le^b^ weakly [24]. For the replication of GII.3 (but not for GII.4) in stem cell-derived human enteroids, human bile is required [25]. Since the distribution of the ABO(H), secretor, and Lewis genotypes is strongly dependent on ethnicity, it is hypothesized that the molecular epidemiology of NoV differs between regions because of host population genetics [26].

The novel GII.17 (subcluster Kawasaki 2014) NoV, the predominant outbreak strain in Japan and China, has recently been reported from several countries across all continents [27–34]. Only two strains of GII.17, from Mongar and Phuntsholing, were detected in Bhutan during our study. Fortunately, they were outside the recent outbreak strains, thus indicating that subcluster Kawasaki 2014 has not yet invaded Bhutan.

Only one Sydney 2012 variant of GII.4 was identified in Bhutan in 2012 [10]; currently, 61.5% of the GII.4 strains in Bhutan are the Sydney 2012 variant, and the rest could not be assigned. In 2010, GII.3 was the predominant NoV in Bhutan [10]. Phylogenetic analysis of other genotypes revealed that the Bhutanese strains were mainly related to strains from neighboring or other Asian countries. The current Bhutanese GII.3 belongs to an independent cluster; these strains were different from those circulating in 2010onseveral nucleotide substitutions, indicating that GII.3 viruses originating from a single GII.3 virus introduction are spreading among these children. Bhutanese strains share a common ancestor with strains from Bangladesh, the USA, and Russia, indicating a common origin of GII.3 circulating in various parts of the world. The Bhutanese GII.4 Sydney 2012 strains were not related to the strains from neighboring countries other than New Zealand and Australia. Although Bhutan is considered a remote location with few connections to other countries, we detected viruses similar to those currently circulating in other parts of the world.

In Bhutan, NoV is prevalent throughout the year with two peaks, one in the colder months, and one in summer. NoV GI was more prevalent during the summer compared with the colder months. We previously reported two peaks in the occurrence of diarrheal cases: one in the colder months and the other during the summer [10]. We recognized then that the peak in the summer was caused by enteric bacteria; further study is needed to determine the contribution of NoV to this peak. The present study has also confirmed our previous observation that the diarrheal peak in the colder months is caused by NoV GII. This is similar to the rotavirus season in Bhutan [35]. Therefore, the peak of diarrhea during the colder months is caused by both rotavirus and NoV GII.

## Conclusions

From the present study, we can conclude that the prevalence of NoV remains one of the important causes of diarrhea among Bhutanese children, and that the strains originating from an ancestor strain of GII.3 that has been spreading has replaced GII.21. Minor genotypes such as GII.6 also showed changes; the circulating subcluster of this genotype changed from subcluster b to a. Phylogenetic analysis showed that other NoV genotypes were similar to the strains circulating worldwide but were primarily related to those in neighboring countries. NoV GII is prevalent in the colder months, while GI is prevalent in the summer. Further surveillance is needed to determine the factors that trigger the dynamics of NoV genotype changes in Bhutanese children. This will help formulate informed policy to control this serious diarrheal disease.

## Supporting information

S1 Table Strains used in the phylogenetic tree. The GenBank accession number, country of origin and year of detection of norovirus GII strains from which the nucleotide sequences of the capsid gene at the C region were used for the construction of the phylogenetic tree.
(DOCX)
