# Supplementary material for: Surveillance of norovirus among children with diarrhea in four major hospitals in Bhutan: Replacement of GII.21 by GII.3 as a dominant genotype
Source: PLoS One. 2017 Sep 14;12(9):e0184826. doi: 10.1371/journal.pone.0184826 (PMC5599041; doi:10.1371/journal.pone.0184826)
Supplement: S1 Table — The GenBank accession number, country of origin and year of detection of norovirus GII strains from which the nucleotide sequences of the capsid gene at the C region were used for the construction of the phylogenetic tree. (DOCX) [file pone.0184826.s001.docx]

# S1 table: The GenBank accession number, country of origin and year of detection of norovirus GII strains from which the nucleotide sequences of the C region of the capsid gene were used for the construction of the phylogenetic tree.

| Strain | Accession No | Country | Year |
| --- | --- | --- | --- |
| Bo/GIII.1/Aba-Z5/2002/HUN(Outgroup) | EU360814 | Hungary | 2002 |
| GII.13 isolate sewage/GII.13/SD2705/2013/CHN | KR107722 | China | 2013 |
| GII.3 isolate sewage/GII.3/SD9002/2013/CHN | KR107689 | China | 2013 |
| Hu/2352/2009/RUS | JX524573 | Russia | 2009 |
| Hu/2669/2011/RUS | JX524570 | Russia | 2011 |
| Hu/30443/2010/VNM | HE716517 | Vietnam | 2010 |
| Hu/C00007915/2010/GBR | HF952128 | United Kingdom | 2010 |
| Hu/C2-230507/2007/SGP | FJ788329 | Singapore | 2007 |
| Hu/Chiba/04-974/2004/JPN | AB220925 | Japan | 2004 |
| Hu/CHN42973-CZ05/2005/CHN | EU072235 | China | 2005 |
| Hu/CMH-N017/2010/THA | KC608635 | Thailand | 2010 |
| Hu/CMH-N164/2011/THA | KC608712 | Thailand | 2011 |
| Hu/Dhaka32/2011/BGD | JX683115 | Bangladesh | 2011 |
| Hu/GII.13/10N4555/2010/NPL | AB810013 | Nepal | 2010 |
| Hu/GII.13/SH20140415-N5/Shanghai/2014/CHN | KP335056 | China | 2014 |
| Hu/GII.16-GII.3/Dhaka53/2012/BGD | JX683114 | Bangladesh | 2012 |
| Hu/GII.17/142700/Shanghai/2014/CHN | KT380915 | China | 2014 |
| Hu/GII.17/C142/1978/GUF | KC597139 | French Guiana | 1978 |
| Hu/GII.17/INCMNSZ-01/2007/MEX | JF970609 | Mexico | 2007 |
| Hu/GII.3/Yunnan/22-YN/2013/CHN | AB924671 | China | 2013 |
| Hu/GII.4 Sydney 2012/Johannesburg_12368/2013/ZAF | KR904504 | South Africa | 2012 |
| Hu/GII.4/20150321_GL06BLPV4/2015/KOR | KU687028 | Korea | 2015 |
| Hu/GII.4/gg-12-08-04/2012/KOR | KM272334 | Korea | 2012 |
| Hu/GII.4/Hunter504D/04O/AUS | DQ078814 | Australia |  |
| Hu/GII.4/New Orleans1805/2009/USA | GU445325 | United States | 2009 |
| Hu/GII.4/Sydney/NSW0514/2012/AUS | JX459908 | Australia | 2012 |
| Hu/GII.4_variant Sydney 2012/NSW317L/2014/AUS | KT239603 | Australia | 2014 |
| Hu/GII.4_variant Sydney 2012/NZ14694/2014/NZL | KT151065 | New Zealand | 2014 |
| Hu/GII.6/Ehime090371/2009/JPN | AB682736 | Japan | 2009 |
| Hu/GII.6/GZ2010-L72/Guangzhou/2010/CHN | JX984949 | China | 2010 |
| Hu/GII.7/1738/2009/USA | GU134965 | United States | 2009 |
| Hu/GII.7/Seoul/0492/2009/KOR | HM635120. | Korea | 2009 |
| Hu/GII.b-GII.3/NSW146P/2007/AUS | GQ845364 | Australia | 2007 |
| Hu/GII.P15-GII.15/RS19603/2011/BRA | KR074189 | Brazil | 2011 |
| Hu/GII.P16_GII.17/Saitama/T87/2002/JPN | KJ196286 | Japan | 2002 |
| Hu/GII.P17_GII.17/CAU-289/2015/KOR | KU561256 | Korea | 2015 |
| Hu/GII.P17_GII.17/CAU-55/2013/KOR | KU561250 | Korea | 2013 |
| Hu/GII.P17_GII.17/Nagano7-1/2014/JPN | LC043139 | Japan | 2014 |
| Hu/GII.P17_GII.17/NSW6016/2014/AUS | KT23964 | Australia | 2014 |
| Hu/GII.P17_GII.17/Saitama5203/2013/JPN | LC043167 | Japan | 2013 |
| Hu/GII/233/2005-2006/JPN | EF630432 | Japan | 2005-2006 |
| Hu/GII/C7-394/2006/KOR | EU249129 | Korea | 2006 |
| Hu/GII/R2U-140208-1/2008/SGP | HM209196 | Singapore | 2008 |
| Hu/Guangzhou/NVgz01/CHN | DQ369797 | China |  |
| Hu/Huzhou122/2012/CHN | KC473545 | China | 2012 |
| Hu/HuzhouN95/2011/CHN | KF048037 | China | 2011 |
| Hu/IDH1021/2008/IND | AB539149 | India | 2008 |
| Hu/GII.4/JG13-420/2013/BTN | LC209758 | Bhutan | 2013 |
| Hu/GII.3/JG13-430/2013/BTN | LC209761 | Bhutan | 2013 |
| Hu/GII.3/JG13-434/2013/BTN | LC209763 | Bhutan | 2013 |
| Hu/GII.2/JG13-496/2014/BTN | LC209769 | Bhutan | 2014 |
| Hu/GII.3/JH-10-003/2010/BTN | LC009621 | Bhutan | 2010 |
| Hu/GII.4/JH-10-0104/2010/BTN | LC009625 | Bhutan | 2010 |
| Hu/GII.3/JH-10-012/2010/BTN | LC009622 | Bhutan | 2010 |
| Hu/GII.3/JH-10-015/2010/BTN | LC009623 | Bhutan | 2010 |
| Hu/GII.3/JH-10-09/2010/BTN | LC009581 | Bhutan | 2010 |
| Hu/GII.7/JH-10-098/2010/BTN | LC009624 | Bhutan | 2010 |
| Hu/GII.3/JH-10-10/2010/BTN | LC009582 | Bhutan | 2010 |
| Hu/GII.3/JH-10-33/2010/BTN | LC009598 | Bhutan | 2010 |
| Hu/GII.3/JH-10-36/2010/BTN | LC009584 | Bhutan | 2010 |
| Hu/GII.4/JH-10-60/2010/BTN | LC009585 | Bhutan | 2010 |
| Hu/GII.3/JH-10-74/2010/BTN | LC009589 | Bhutan | 2010 |
| Hu/GII.4/JH-11-0134/2011/BTN | LC009627 | Bhutan | 2011 |
| Hu/GII.21/JH-11-023/2011/BTN | LC009588 | Bhutan | 2011 |
| Hu/GII.6/JH-11-164/2011/BTN | LC009608 | Bhutan | 2011 |
| Hu/GII.6/JH-11-188/2011/BTN | LC009609 | Bhutan | 2011 |
| Hu/GII.21JH-11-207/2011/BTN | LC009610 | Bhutan | 2011 |
| Hu/GII.6/JH-11-210/2011/BTN | LC009611 | Bhutan | 2011 |
| Hu/GII.6/JH-11-236/2011/BTN | LC009599 | Bhutan | 2011 |
| Hu/GII.6/JH-11-239/2011/BTN | LC009628 | Bhutan | 2011 |
| Hu/GII.2/JH-11-268/2011/BTN | LC009580 | Bhutan | 2011 |
| Hu/GII.6/JH-11-279/2011/BTN | LC009601 | Bhutan | 2011 |
| Hu/GII.5/JH-11-307/2011/BTN | LC009612 | Bhutan | 2011 |
| Hu/GII.2/JH-11-317/2011/BTN | LC009603 | Bhutan | 2011 |
| Hu/GII.6/JH-11-321/2011/BTN | LC009629 | Bhutan | 2011 |
| Hu/GII.20/JH-11-331/2011/BTN | LC009604 | Bhutan | 2011 |
| Hu/GII.2/JH-11-348/2012/BTN | LC009613 | Bhutan | 2012 |
| Hu/GII.4/JH-11-352/2012/BTN | LC009600 | Bhutan | 2012 |
| Hu/GII.6/JH-11-372/2012/BTN | LC009578 | Bhutan | 2012 |
| Hu/GII.2/JH-11-381/2012/BTN | LC009616 | Bhutan | 2012 |
| Hu/GII.2/JH-11-406/2012/BTN | LC009620 | Bhutan | 2012 |
| Hu/GII.2/JH-11-414/2012/BTN | LC009602 | Bhutan | 2012 |
| Hu/GII.6/JH-11-417/2012/BTN | LC009587 | Bhutan | 2012 |
| Hu/GII.6/JH13 154/2013/BTN | LC209711 | Bhutan | 2013 |
| Hu/GII.13/JH13-005/2013/BTN | LC209686 | Bhutan | 2013 |
| Hu/GII.3/JH13-080/2013/BTN | LC209689 | Bhutan | 2013 |
| Hu/GII.3/JH13-082/2013/BTN | LC209690 | Bhutan | 2013 |
| Hu/GII.3/JH13-100/2013/BTN | LC209693 | Bhutan | 2013 |
| Hu/GII.4/JH13-107/2013/BTN | LC209695 | Bhutan | 2013 |
| Hu/GII.3/JH13-111/2013/BTN | LC209696 | Bhutan | 2013 |
| Hu/GII.3/JH13-114/2013/BTN | LC209697 | Bhutan | 2013 |
| Hu/GII.3/JH13-132/2013/BTN | LC209700 | Bhutan | 2013 |
| Hu/GII.4/JH13-145/2013/BTN | LC209706 | Bhutan | 2013 |
| Hu/GII.3/JH13-151/2013/BTN | LC209708 | Bhutan | 2013 |
| Hu/GII.6/JH13-156/2014/BTN | LC209712 | Bhutan | 2014 |
| Hu/GII.3/JH13-157/2014/BTN | LC209713 | Bhutan | 2014 |
| Hu/GII.3/JH13-159/2014/BTN | LC209714 | Bhutan | 2014 |
| Hu/GII.3/JH13-162/2014/BTN | LC209715 | Bhutan | 2014 |
| Hu/GII.3/JH13-179/2014/BTN | LC209719 | Bhutan | 2014 |
| Hu/GII.22/JH13-236/2014/BTN | LC209726 | Bhutan | 2014 |
| Hu/GII.3/JH13-240/2014/BTN | LC209728 | Bhutan | 2014 |
| Hu/GII.15/JM13-106/2013/BTN | LC209770 | Bhutan | 2013 |
| Hu/GII.4/JM13-126/2013/BTN | LC209774 | Bhutan | 2013 |
| Hu/GII.3/JM13-133/2014/BTN | LC209777 | Bhutan | 2014 |
| Hu/GII.3/JP13-715/2013/BTN | LC209754 | Bhutan | 2013 |
| Hu/GII.17/JP13-716/2013/BTN | LC209755 | Bhutan | 2013 |
| Hu/GII.15/JP13-718/2014/BTN | LC209756 | Bhutan | 2014 |
| Hu/JP41/2012/JPN | KF145149 | Japan | 2012 |
| Hu/Leverkusen267/2005/GER | EU424333 | Germany | 2005 |
| Hu/Luckenwalde591/2002/GER | EU373815 | Germany | 2002 |
| Hu/GII.4/M-1298/2013/BTN | LC209738 | Bhutan | 2013 |
| Hu/GII.4/M-1348/2014/BTN | LC209740 | Bhutan | 2014 |
| Hu/GII.4/M-1479/2014/BTN | LC209743 | Bhutan | 2014 |
| Hu/GII.4/M-1564/2014/BTN | LC209744 | Bhutan | 2014 |
| Hu/GII.4/M-1604/2014/BTN | LC209748 | Bhutan | 2014 |
| Hu/GII.4/M-1678/2014/BTN | LC209751 | Bhutan | 2014 |
| Hu/GII.4/M-1693/2014/BTN | LC209752 | Bhutan | 2014 |
| Hu/GII.13/M-937/2013/BTN | LC209730 | Bhutan | 2013 |
| Hu/GII.4/M-941/2013/BTN | LC209731 | Bhutan | 2013 |
| Hu/GII.13/M-942/2013/BTN | LC209732 | Bhutan | 2013 |
| Hu/GII.15/M-984/2013/BTN | LC209735 | Bhutan | 2013 |
| Hu/GII.13/M-993/2013/BTN | LC209736 | Bhutan | 2013 |
| Hu/Melksham/1994/GBR | X81879 | United Kingdom | 1994 |
| Hu/Milwaukee009/2010/USA | JN565063 | United States | 2010 |
| Hu/NLV/IF1998/2003/IRQ | AY675554 | Iraq | 2003 |
| Hu/NLV/J23/1999/USA | AY130762 | United States | 1999 |
| Hu/NLV/Seacroft/1990/GBR | AJ277620 | United Kingdom | 1990 |
| Hu/NoV/GII.22/YURI/JPN | AB083780 | Japan |  |
| Hu/NoV/Katrina-17/2005/USA | DQ438972 | United States | 2005 |
| Hu/OC05114/2005/Osaka-JPN | AB662851 | Japan | 2005 |
| Hu/OC08079/2008/Osaka-JPN | AB662859 | Japan | 2008 |
| Hu/Pune/PC01/2005/IND | EU921330 | India | 2005 |
| Hu/Seoul1913/2012/KOR | JX439905 | Korea | 2012 |
| Hu/GII.P22 GII.5/T49/2001/Saitama-JPN | AB112315 | Japan | 2001 |
| Hu/V1628/2006/IND | AB453773 | India | 2006 |
| Hu/V1656/2006/IND | AB453774 | India | 2006 |
| Hu/YO284/GII.P21 GII.21/2007/Kawasaki-JPN | KJ196284 | Japan | 2007 |
| Hu/Zuerich/P7d1/2008/CHE | GQ266696 | Switzerland | 2008 |
| JB031230054/Luohu/OB/12/2012/CHN | KJ955492 | China | 2012 |
| Hu/GII.3/CHDC4671/1979/USA | HM072042 | USA | 1979 |
| Hu/GII.3/CHDC2005/1975/USA | HM072045 | USA | 1975 |
| Hu/GII.3/CHDC4031/1988/USA | HM072044 | USA | 1088 |
| NLV/Lionville/247/1993/USA | AF414411 | USA | 1993 |
| Hu/Maizuru/010524/2001/JPN | EF547399 | Japan | 2001 |
| NLV/Brattleboro/321/1995/USA | AF414415 | USA | 1995 |
| wastewater/NW_Oct11_1077_1/2011/ZAF | KC495681 | South Africa | 2011 |
| water/GII.21/W8/2013/BTN | LC009630 | Bhutan | 2013 |
